# Correlation of HPV16 Gene Status and Gene Expression With Antibody Seropositivity and TIL Status in OPSCC

**DOI:** 10.3389/fonc.2020.591063

**Published:** 2021-01-26

**Authors:** Adrian von Witzleben, Eve Currall, Oliver Wood, Lindsey Chudley, Oluyemisi Akinyegun, Jaya Thomas, Kaïdre Bendjama, Gareth J. Thomas, Peter S. Friedmann, Emma V. King, Simon Laban, Christian H. Ottensmeier

**Affiliations:** ^1^ CRUK and NIHR Experimental Cancer Medicine Center & School of Cancer Sciences, Faculty of Medicine, University of Southampton, Southampton, United Kingdom; ^2^ Department of Otorhinolaryngology, Head & Neck Surgery, University of Ulm, Ulm, Germany; ^3^ Southampton University Hospitals NHS Foundation Trust, Southampton, United Kingdom; ^4^ Department Affaires Médicinales, Research, Project, Transgene SA, Illkirch, France; ^5^ Department of Otorhinolaryngology, Head & Neck Surgery, Poole Hospital, Poole, United Kingdom; ^6^ Liverpool Head and Neck Centre, Institute of Translational Medicine, Department of Molecular & Clinical Cancer Medicine, University of Liverpool, Liverpool, United Kingdom

**Keywords:** oropharyngeal squamous cell carcinoma, human papillomavirus 16, antibody isotype, gene expression, immune response

## Abstract

**Introduction:**

Human papillomavirus 16 (HPV16) is the main cause of oropharyngeal squamous cell carcinoma (OPSCC). To date, the links between HPV16 gene expression and adaptive immune responses have not been investigated. We evaluated the correlation of HPV16 DNA, RNA transcripts and features of adaptive immune response by evaluating antibody isotypes against E2, E7 antigens and density of tumor-infiltrating lymphocytes (TIL).

**Material and Methods:**

FFPE-tissue from 27/77 p16-positive OPSCC patients was available. DNA and RNA were extracted and quantified using qPCR for all HPV16 genes. The TIL status was assessed. Immune responses against E2 and E7 were quantified by ELISA (IgG, IgA, and IgM; 77 serum samples pre-treatment, 36 matched post-treatment).

**Results:**

Amounts of HPV16 genes were highly correlated at DNA and RNA levels. RNA co-expression of all genes was detected in 37% (7/19). E7 qPCR results were correlated with higher anti-E7 antibody (IgG, IgA) level in the blood. Patients with high anti-E2 IgG antibody (>median) had better overall survival (p=0.0311); anti-E2 and anti-E7 IgA levels had no detectable effect. During the first 6 months after treatment, IgA but not IgG increased significantly, and >6 months both antibody classes declined over time. Patients with immune cell-rich tumors had higher levels of circulating antibodies against HPV antigens.

**Conclusion:**

We describe an HPV16 qPCR assay to quantify genomic and transcriptomic expression and correlate this with serum antibody levels against HPV16 oncoproteins. Understanding DNA/RNA expression, relationship to the antibody response in patients regarding treatment and outcome offers an attractive tool to improve patient care.

## Introduction

Head and neck squamous cell carcinoma (HNSCC) is the sixth commonest cancer in the western world (1, 2). 25% of all cases of HNSCC are associated with human papillomavirus (HPV) and 60% of oropharyngeal squamous cell carcinomas (OPSCC) are HPV-driven (3, 4). Infection with a high-risk HPV sub-type can result in the development of OPSCC in individuals irrespective of classical risk factors such as tobacco use and alcohol consumption (5). 

The coding regions of the HPV genome consist of an early region (E) with six open reading frames (ORF) E1, E2, E4, E5, E6, E7, and a late region (L) with the ORFs, L1 and L2. The viral oncoproteins E6 and E7, interact with tumor suppressors p53 and Rb, respectively, inactivating their protective function and resulting in aberrant cell cycle control (6). As a result of the functional inactivation of pRb by E7, p16 is upregulated; hence high expression of p16 is often used as a surrogate diagnostic marker for HPV16 (7). E2 has important regulatory function in E6 and E7 gene transcription (8) and viral replication, this function can be disrupted through integration (9). The viral oncoprotein E5 can reduce cell surface expression of the HLA class I, thereby promoting tumor escape from immune control by CD8^+^ T cells (10). Nonetheless, patients with HPV-positive (HPV^pos^) OPSCC have a better prognosis in comparison to those with HPV-negative (HPV^neg^) OPSCC (5). This is most likely attributable to the patient’s anti-tumor immune response and is independent of individual treatment regimens (11, 12). The immunological ‘visibility’ to T cells can be assessed by counting the number of tumor-infiltrating lymphocytes (TILs), higher TIL-density correlates with better survival. In contrast, patients with HPV^pos^ OPSCC but a low TIL-density have a disease-related survival resembling that of HPV16^neg^ OPSCC (13). HPV16 antigens can also be recognized by B cells. Seropositivity to the early genes E1, E2, E4, E6, E7, and L1 has been described as a serological markers for the presence of HPV16^pos^ OPSCC (14, 15). HPV16^pos^ OPSCC patients show increased levels of E6 and E7 antibody in the blood independent of the viral load (16). Humoral immune responses also link to outcome: in OPSCC, seropositivity to HPV16 E6 is associated with a 68% reduction in the risk of death (17). Survival benefit was also reported in OPSCC patients who were found to be seropositive to HPV16 E1, E2, or E6 (18).

Systematic analyses of HPV16 genes at the DNA and RNA level, and their correlation to TIL status and humoral immune responses have not yet been published; it further remains unclear how often antibody class switching occurs in HPV^pos^ HNSCC patients.

In this study, we aimed to evaluate the expression of HPV16 genes at a genomic and transcriptomic level, using a qPCR assay. This helps to understand, which genes of HPV16 genome are present in the tumor at the DNA level, and which of them are transcribed in HNSCC. The data were related to the presence of IgG, IgA and IgM antibodies against the HPV16 E2 and E7 antigens, as determined by ELISA. We used paired serum samples before and after treatment, to measure IgG and IgA antibody response to HPV16 E2 and E7 to determine whether treatment influences the antibody response and how the removal of the cancer as the source of antigen affects antibody levels over time. Additionally, we assessed the TIL status for those tumors and related this to the HPV16 expression and antibody responses.

## Material and Methods

### Study Ethics

The study given UK Medical Research and Ethics Committee (MREC 09/H0501/90) and institutional approval at Southampton University Hospitals NHS Foundation Trust, Southampton, UK. Written informed consent was obtained from all patients.

### Patient Cohort

Patient samples were collected between 2011 and 2018 (**Supplementary Table 1**). Perioperative serum samples (n=77) were selected from patients with p16^+^ oropharyngeal squamous cell carcinoma (OPSCC). Stored serum samples were available for all patients, 73/77 patients had pre-treatment samples, 40/77 patients had post-treatment samples, 36/77 patients had both pre and post-treatment samples available.

Where available, formalin fixed, paraffin embedded (FFPE) material (n=27) from the primary tumor were retrieved from pathology archives. Snap-frozen tissue samples were available for 4/77 patients. TNM was re-staged according to the 8^th^ edition (AJCC).

### DNA, RNA Extraction From FFPE Tissue and RT-PCR

DNA and RNA were extracted from FFPE tissue in accordance to the manufacturer’s protocol (Maxwell^®^ RSC DNA FFPE Kit, AS1450, Maxwell^®^ RSC RNA FFPE Kit, AS1440, Promega, Southampton, UK). The concentrations of DNA and RNA were measured using the Nanodrop 2000 (Thermo Scientific, Waltham, MA, USA). All samples were diluted to a final concentration of 10 ng/µl (DNA), 20 ng/µl (RNA). 2 µl RNA was used for reverse transcription (20 µl, Superscript III first-strand synthesis system, Thermo Scientific, Waltham, MA, USA).

### qPCR for HPV16

Quantitative polymerase chain reaction (qPCR) was performed (GoTaq qPCR systems assay, Promega, Southampton, UK) using 1 µl each of DNA (10ng) and 1 µl of cDNA. Samples were loaded onto 384 well plates in triplicates, read on the Applied Biosystems 7900HT workstation and analyzed using SDS 2.3 software (Thermo Fisher, Waltham, MA, USA). The qPCR settings on the workstation were adjusted according to the manufacturer’s protocol for the GoTaq^®^ assay.

Published primer pairs for all HPV16 genes used are shown in **Table 1** (19–26). Primer pair annealing to the HPV16 genome NC_001526.4 was confirmed using Primer-BLAST (27, 28). Primer pairs were confirmed *in silico* to be specific for HPV16 and to not bind to other high-risk virus genomes. Specificity was verified using NCBI without detecting any unintended templates of common human viruses (taxid: 10239). Four E5 primer pairs were evaluated as we could not generate a PCR product using the initial primer pair [Paolini et al. (22)], because of missing genomic complementary sequence.

**Table 1:** Primer pairs (custom DNA primer, Sigma-Aldrich) used for qPCR with GoTag qPCR kit (Promega) and SYBR settings including their sequence in 5’-3’ direction, the length of the PCR product, location on the HPV16 genome (NC_001526.4), and the citation to the originate paper.

Gene	Forward Primer 5’->3’	Reverse Primer 5’->3’	Size (bp)	Location (HPV16 genome)	Citation
E1	AGTAGAGCTGCAAAAAGGAGATTA	CTGACTACATGGTGTTTCAGTCTC	123	355-454	Nilsson et al. (19)
E2	AACGAAGTATCCTCTCCTGAAATTATTAG	CCAAGGCGACGGCTTTG	82	2498-2563	Peitsaro et al. (20)
E4	GACTATCCAGCGACCAAGATCAG	CTGAGTCTCTGTGCAACAACTTAGTG	77	2599-2650	Egawa et al. (21)
E5	GCGACGTGAGAGCAACG	AGGGGTTTCCGGTGTCTGG	na	Not found	Paolini et al. (22)
E5	GCATCCACAACATTACTGGCG	GTAGACACAGACAAAAGCAGCGG	95	3004-3076	Um et al. (23)
E5	CTTTGCTTTTGTGTGCTTTTGTGTG	AAAGCGTGCATGTGTATGTATTAAA	192	3034-3201	Sahab et al. (24)
E5	ATGACAAATCTTGATACTGCA	AATGATGTGTATGTAGACACAG	125	2986-3089	Campo et al. (25)
E6	GAGAACTGCAATGTTTCAGGACC	TGTATAGTTGTTTGCAGCTCTGTGC	81	7136-7192	Peitsaro et al. (20)
E7	AAGTGTGACTCTACGCTTCGGTT	GCCCATTAACAGGTCTTCCAAA	78	7781-7837	Wang-Johanning et al. (26)
L1	TTAGGTGTGGGCATTAGTGG	TCCCCTATAGGTGGTTTGCA	164	5111-5255	Nilsson et al. (19)
L2	GACCCTGCTTTTGTAACCACTC	ATGCTGGCCTATGTAAAGCAAC	166	4087-4231	Nilsson et al. (19)
ß-actin	TCACCCACACTGTGCCCATCTACGA	CAGCGGAACCGCTCATTGCCAATGG	295	n.a.	Wang-Johanning et al. (26)

The expression values of the samples (s) were calculated with the modified ΔΔCt relative expression method (29, 30). The median Ct(cycle threshold)-value of the gene of interest (GOI) was normalized against the reference mean (ß-actin) (26). ΔΔCt relative values were calculated of the positive control (pc) and subtracted from the total number of cycles to obtain positively correlation values:

$$40-\Delta \Delta Ct\left(GOI\right)=40-\left(\left(meanCt{\left[GOI\right]}_{s}-meanCt{\left[REF\right]}_{s}\right)-\left(meanCt{\left[GOI\right]}_{pc}-meanCt{\left[REF\right]}_{pc}\right)\right)$$

This is a semi-quantitative method; therefore, the relative values are reported as “amount” or “levels” of DNA and of RNA expression.

The RNA qPCR was not successful in a subgroup of samples. We were not able to detect the ß-actin gene, predominantly from older FFPE pathological blocks (>5 years).

### HPV16 Viral Genome Level Analysis

RNA was extracted from snap-frozen tissue of four patients (Maxwell^®^ RSC simplyRNA Tissue Kit(AS1340), Promega, Southampton, UK). RNA concentration and quality were analyzed using the RNA Nano Kit for the 2100 Bioanalyzer System (Agilent Technologies, Santa Clara, CA, USA). RNA-sequencing was undertaken by Edinburgh Genomics (University of Edinburgh, Edinburgh, UK). An automated TruSeq stranded mRNA-seq library preparation from total RNA and the NovaSeq sequencing-system was used (100 bp paired-end; 1,750M+1,750M reads, Illumina, San Diego, CA, USA). This RNA sequencing dataset was generated in an independent collaboration with Transgene, and the fact that Transgene funded the RNA sequencing for four cases of the study, did not influence the study design, execution, and results interpretation.

The “viral genome level analysis module” of the bioinformatics pipeline viGen (31) was used to align the FASTQ files against the human reference genome (hg19), filter out human RNA-sequences and map un-aligned reads against the viral reference. Access to the raw and processed data of this RNA sequencing set is possible *via* the gene expression omnibus (GEO accession number: GSE160008).

### Immunohistochemistry

p16 IHC on formalin-fixed, paraffin-embedded (FFPE) tumor tissue was performed as part of the routine diagnostics process (**Supplementary Table 1**). Additional IHC was performed on FFPE tissue using standard protocols for the automated platforms Dako PT Link for Heat Induced Epitope Retrieval and Dako Autostainer 48S Link (Agilent, Santa Clara, CA, USA) with staining of tumor-infiltrating lymphocytes using anti-CD8 antibodies (Anti-Human CD8, Clone C8, 144B, Dako, concentration: 1:100, Agilent, Santa Clara, CA, USA). An unsuccessful immunohistochemical approach for HPV16 E2 and E7 was performed with following antibodies [Anti-HPV16 E2, NBP2-53115, NovusBio (no literature), Centennial, CO, USA; Anti-HPV16 E7, ab20191, Abcam, Cambridge, UK (32); Anti-HPV16 E7 (716–325), sc-51951, Santa Cruz Biotechnology, Dallas, TX, USA (33)]. Different concentrations and antigen retrieval techniques revealed still unspecific staining.

Frequency and density of CD8 positive T cells were determined as previously described (34, 35). One case was excluded in the further IHC analysis due to non-specific IHC staining.

### Expression and Purification of HVP16 E2 and E7 Protein

HPV16 E2 and E7 protein were produced in the protein core facility of the CRUK Experimental Cancer Sciences Center, Faculty of Medicine, Southampton University.

Control-GST and GST-E2 were expressed as soluble proteins in bacteria, purified on GSTrap column (17528101, GE Healthcare, Chicago, IL, USA) and dialyzed in PBS. GST-E7 was expressed as an insoluble protein in bacteria. The protein was first purified under denaturing conditions in the presence of urea on a HiTrap Q HP anion exchange column (17115301, GE Healthcare, Chicago, IL, USA) and dialyzed in buffer without urea. The dialyzed material was then purified under native conditions on a HiTrap Q HP anion exchange column prior to gel filtration in PBS.

The following plasmids were used: p3187 HPV-16 E2 was a gift from Peter Howley (Addgene plasmid#10846; http://n2t.net/addgene:10846;RRID : Addgene_10846) (36). pGEX2T E7 was a gift from Karl Munger (Addgene plasmid#13634; http://n2t.net/addgene:13634;RRID : Addgene_13634) (37). Different E6 protein expression and purification methods failed, as non-reliable ELISA results were detected.

### ELISA

A standard ELISA was performed in triplicates including a standard curve and negative control. Plates were coated with GST HPV16 E2 and E7 with a working concentration of 2µg/mL at 4°C overnight. Total IgG (goat anti-human (Fc), HRP, A0179, Sigma, St. Louis, MO, USA), IgA (Goat anti-human IgA (Heavy chain), HRP, PA1-74395), and IgM (Goat anti-human IgM (Heavy chain), HRP, A18835, Thermo Scientific, Waltham, MA, USA) were used as secondary antibodies. The Multiskan FC Microplate Photometer was used to analyze the plates, and OD values were exported using the SkanIt software (Thermo Scientific, Waltham, MA, USA).

A standard curve was generated by seven steps of doubling dilutions starting at a concentration of 1:100 using a pool of positive sera. Antibody titers were measured in arbitrary units (AU) using this standard curve. The negative control serum pool was used to set the positive cut-off point (mean+1.645 standard deviations).

### Analysis and Statistics

The statistical evaluations were undertaken and graphed using Microsoft Excel (version 16.33) and GraphPad Prism (version 8.4.2). Nonparametric unpaired (Mann-Whitney test) or paired tests (Wilcoxon signed-rank test) were used. Correlations are reported using the nonparametric Spearman’s correlation coefficient. An r-value <0.3 was deemed as very weak, 0.3–<0.5 as weak, 0.5–0.7 moderate and >0.7 as strong (38). The survival analysis was performed using the Kaplan-Meier estimator and a Mantel-Cox log-rank test to compare survival curves. P-values less than 0.05 was considered statistically significant.

## Results

### Patient Summary

Our clinical cohort of 77 patients included only p16^+^ oropharyngeal tumors (patient characteristics shown in **Table 2**). Most of the tumors were histologically non-keratinizing squamous cell carcinoma (58/77, 75%). Higher tumor stage was associated with poor survival (p=0.0133, **Figure S1**). The patient’s treatment is summarized in **Table 2**. Three patients had after primary CRT/RT a surgical salvage (only preoperative serum samples were evaluated). The median follow-up time was 4 years and 5 months (Overall survival: 2-year: 94%, 5-year: 85.3%). No significant difference in survival according to gender, age, tumor site or nodal status was found.

**Table 2:** Summary of the patient characteristics.

Descriptive Statistics		n=	Percentage
Patients	All	77	100%
			
Gender	Male	67	87%
	Female	10	13%
			
Age	Median (years)	56	
	Range (years)	35-79	
			
Tumor	Oropharynx	77	100%
	Base of Tongue	28	36%
	Tonsil	49	64%
			
T status	T1	15	19%
	T2	36	47%
	T3	19	25%
	T4	7	9%
			
N Status	N0	6	8%
	N1	14	18%
	N2	53	69%
	N3	4	5%
			
Treatment	Surgery +/- adj. CRT/RT	23	30%
	CRT/RT	51	66%
	CRT/RT + Surgery	3	4%

### Analysis of HPV16 DNA and RNA

The HPV16 genes E1, E2, E4, E5, E6, E7, L1, and L2, were assessed using qPCR assays on DNA and cDNA from 27 p16^+^ OPSCC patients. We could not detect the complementary sequence in the HPV16 genome for the primer pair published by Paolini et al. (22) using primer-BLAST (28). This primer pair was not used in further analyses. Additional three published E5 primer sets were evaluated (**Supplementary Table 1**). A concordant correlation of E5 DNA with the other HPV16 genes was observed using these primer pairs (**Figure S2**). For further analysis we chose to proceed with the E5 primer pair which had previously been used in HNSCC (Um et al.) (23).

**Figure 1:**
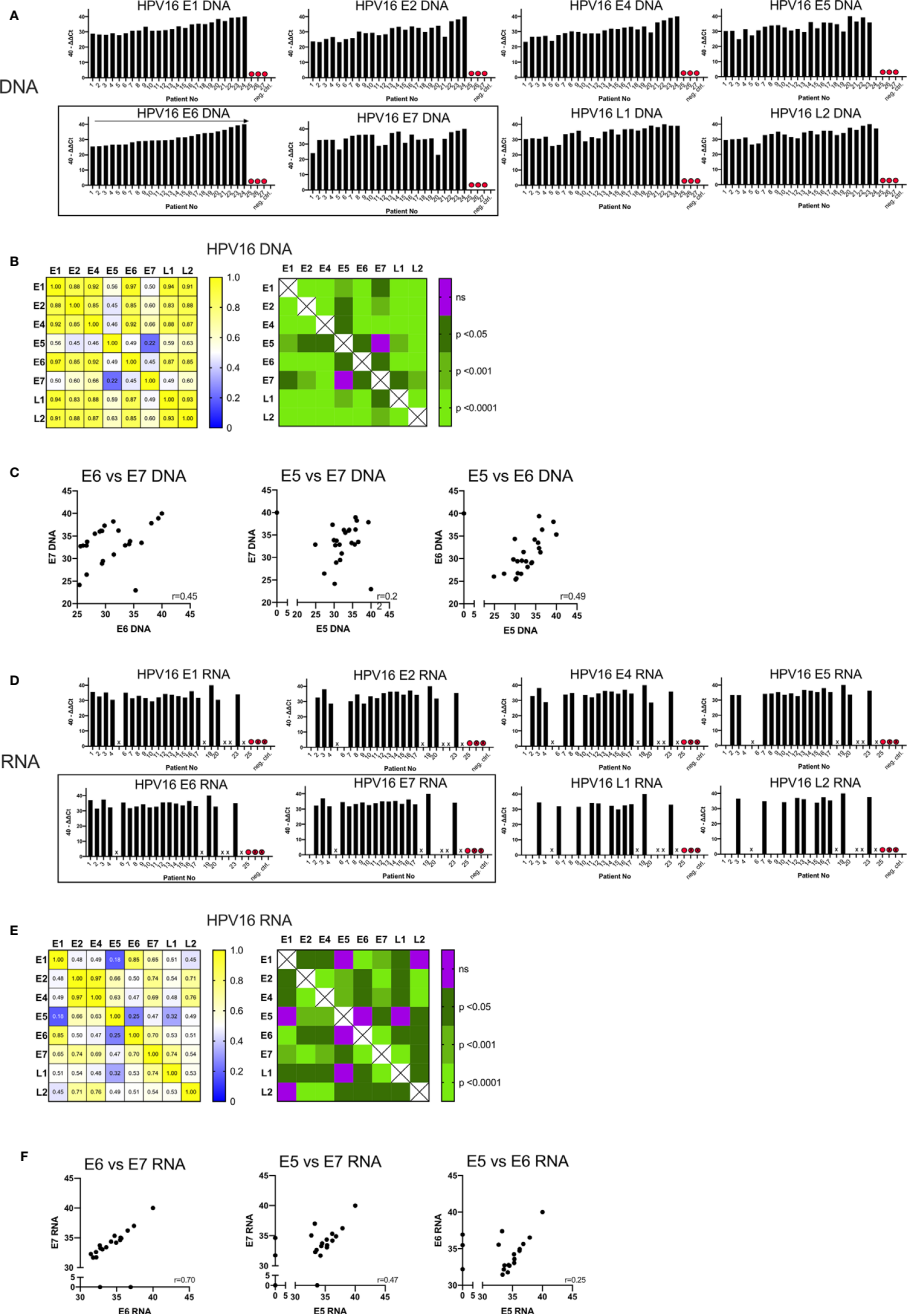
Graphs showing the qPCR data of 27 p16^+^ HNSCC patients analyzed. **(A)** Bar graph displaying 40-ΔΔCT values of DNA for all HPV16 genes of all patients including the blank and negative control (p16 and HPV16 negative non oropharyngeal HNSCC) ordered by the amount of E6 DNA. HPV16^neg^ false p16 positive cases are marked with a red dot. **(B)** The correlation matrix on the left shows the Spearman’s correlation coefficients (r) of each HPV16 gene DNA quantity compared to each other. The right matrix displays the corresponding significance levels. **(C)** Scatter plot displaying individual correlations between the DNA levels of the oncogenic genes E5, E6, and E7. **(D)** Bar graph displaying 40-ΔΔCT values of RNA for all HPV16 genes of all patients including the blank and negative control (p16 and HPV16 negative non oropharyngeal HNSCC) ordered by the amount of E6 DNA **(A)**. HPV16^neg^ false p16 positive cases are marked with a red dot and not analyzable cases are not labelled on the x-axis and are marked with a cross. **(E)** The correlation matrix on the left shows the Spearman’s correlation coefficients (r) of each HPV16 gene RNA expression compared to each other. The right matrix displays the corresponding significance levels. **(F)** Scatter plot displaying individual correlations between the RNA expression of the oncogenic genes E5, E6, and E7.

The quantity of DNA identified for the early and late genes is shown in **Figure 1A**, expressed as 40-ΔΔCt values and ordered by the amount of DNA of the oncogenic E6. In three patients (Case 25, 26, 27) no DNA was detected for any HPV16 gene; these cases were excluded from further analysis and classified as false positive (HPV-status) p16 IHC results.

In the remaining 24 samples, HPV16 DNA was present (40-ΔΔCT range: 22.96-40) (**Figure 1A**). 40-ΔΔCT was highly consistent for E6, E1, E2, E4, L1, and L2 within any individual patient. By contrast, the 40-ΔΔCT detection thresholds of DNA for E7 and E5 genes were less correlated with the other HPV16 genes (in particular case 3). No E5 DNA was detected in case 24 (**Figure 1A**), despite this patient having the highest 40-ΔΔCT values for the other HPV16 genes.

The correlation matrix in **Figure 1B** shows the Spearman’s correlation coefficients (r-values) and the respective p-values. The analysis showed a high correlation of E6 DNA to that of the other genes (strongest for E1 and E6, r=0.97, p<0.0001). In contrast, the amounts of E7 DNA were less correlated with other HPV genes, especially with E5 DNA (r=0.22, not significant) (**Figure 1B**). This is consistent with the visual impression in **Figure 1A**. E7 and E5 were only modestly correlated with the 40-ΔΔCT values from other HPV16 genes; the r-values ranged from 0.45 to 0.66 (p<0.05, **Figure 1B**). A highly significant correlation (r>0.85, p<0.001) was observed for DNA amounts among the early genes E1, E2, E4, and E6 with each other and the late genes L1 and L2 (**Figure 1B**). Per-patient correlations between the DNA levels of the oncogenic genes E5, E6, and E7 are displayed in a scatterplot in **Figure 1C** with an r-value of 0.45 for E6 vs E7.

To study transcription of the HPV16 genes, we performed qPCR analysis on cDNA. In seven cases (5, 18, 21, 22, 24, 26, and 27) the quality and amount of RNA was too low to detect the housekeeping gene, β-actin and these are marked with a cross on **Figure 1D**. As expected in case 25, (previously identified as HPV16 negative) we did not detect HPV16 RNA. Overall, a total of 19 samples yielded product in the qPCR RNA expression analysis and we detected 40-ΔΔCt values ranging from 28.6–40.

RNA expression of all HPV16 genes were found in 7/19 (37%) patients. In five patients we detected all early genes but only one late gene (26%); in two cases we found all early genes but no late genes (11%) (**Figure 1D**). In case 4 we found RNA for all early genes except E5; and in case 20 we found no E7 RNA despite the detection of DNA. Overall, expression of RNA for late genes was detected in 11/19 cases (58%) for L1, 10/19 cases (53%) for L2, and seven cases had a co-expression of L1 and L2 (37%).

Using Spearman’s correlation coefficient to assess HPV16 genes (**Figure 1E**), E6 transcripts were highly correlated with those for E1 (r=0.85, p<0.0001) and E7 (r=0.7, p<0.0001), while E7 transcripts were highly correlated with those for E2/L1 (r=0.74, p<0.0001). Poor correlation was shown between E5/E6 (r=0.25, not significant), E5/E7 (r=0.47, p<0.05) and E5/E1 transcripts (r=0.18, not significant). However, E5 RNA was highly correlated with E2 (r=0.66, p<0.001) and E4 RNA (r=0.63, p<0.001). **Figure 1E** also shows the strongest correlation between the transcripts for E2/E4 (r=0.97).

Per-patient correlations between the RNA expression for the oncogenic genes E5, E6 and E7 are shown in **Figure 1F**. For both E6/E7 and E5/E7 the correlation between RNA expression of those oncogenes has a moderate (r=0.47) and high (r=0.70) correlation value respectively. Whereas the E5/E6 correlation was low and not statistically significant.

**Figure 2:**
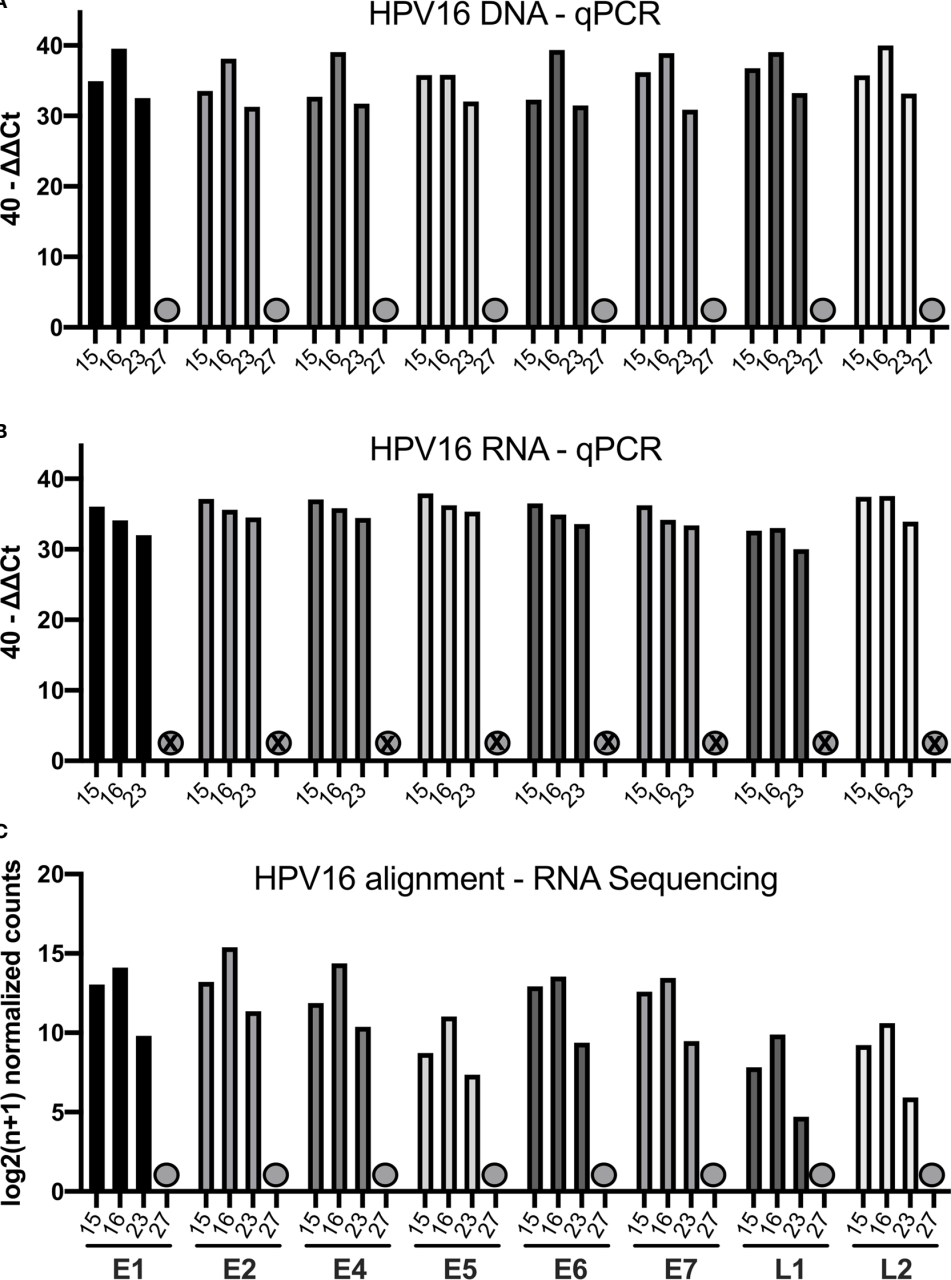
Bar graphs showing the qPCR and RNA-sequencing data of four selected patients (15, 16, 23, 27). **(A)** Bar graph displaying 40-ΔΔCT values of DNA for all HPV16 genes of those patients. The HPV16^neg^ (false p16 positive case 27) is marked with a grey dot. **(B)** Bar graph showing the corresponding 40-ΔΔCT values of RNA for the respective patients **(C)** Bar graph showing the log2(n+1) normalized counts for the HPV16 genome using RNA-sequencing data of respective patients.

We quantified the HPV16 viral transcripts in 4 cases (15, 16, 23, and 27) using RNA-sequencing and compared this to the qPCR results. The 40-ΔΔCt values assessed by qPCR for DNA and RNA are displayed together with the HPV16 genome alignment using RNA-sequencing data in **Figure 2**. Consistent with data from qPCR, case 27 did not have any expressed HPV16 genes detected by RNA-sequencing either (previously identified as HPV16^neg^). The other three cases showed high amount for all HPV16 genes and RNA transcripts assessed by qPCR as well as by using RNA-sequencing alignment.

### Correlation of IgG and IgA Antibodies to HPV16 E2 and E7

Total IgM, IgG, and IgA antibody responses to E2, E6, and E7 antigens were evaluated using ELISA. E6 protein showed unreliable and inconsistent results, most likely due to problems in protein folding in the protein expression system (data not shown). 3/73 pre-treatment samples (4%) contained strongly detectable IgM antibodies against E2 and E7 simultaneously, perhaps reflecting recent re-exposure of HPV16. Interestingly, the HPV^neg^ case 25 had low levels of circulating E2 IgG (AU=0.2) and E7 IgA (AU=0.8), likely reflecting an unrelated, cleared infection with HPV16. The other two HPV^neg^ cases (26 and 27), had no detectable antibodies to E2 and E7.

Of the remaining 70 pre-treatment serum samples, 46 were positive for E2 IgG, 56 for E7 IgG, 28 for E2 IgA, and 63 for E7 IgA. Thirty-nine cases were positive for E2 and E7 IgG, while 24 had positive ELISA results for E2 and E7 IgA. Positive results for all different antigen-antibody combinations were found in 20 cases. We assessed the relationship between IgA and IgG antibody levels (including the post-treatment samples; **Figure S3**). The antibody levels were correlated with weak to high correlation values.

**Figure 3:**
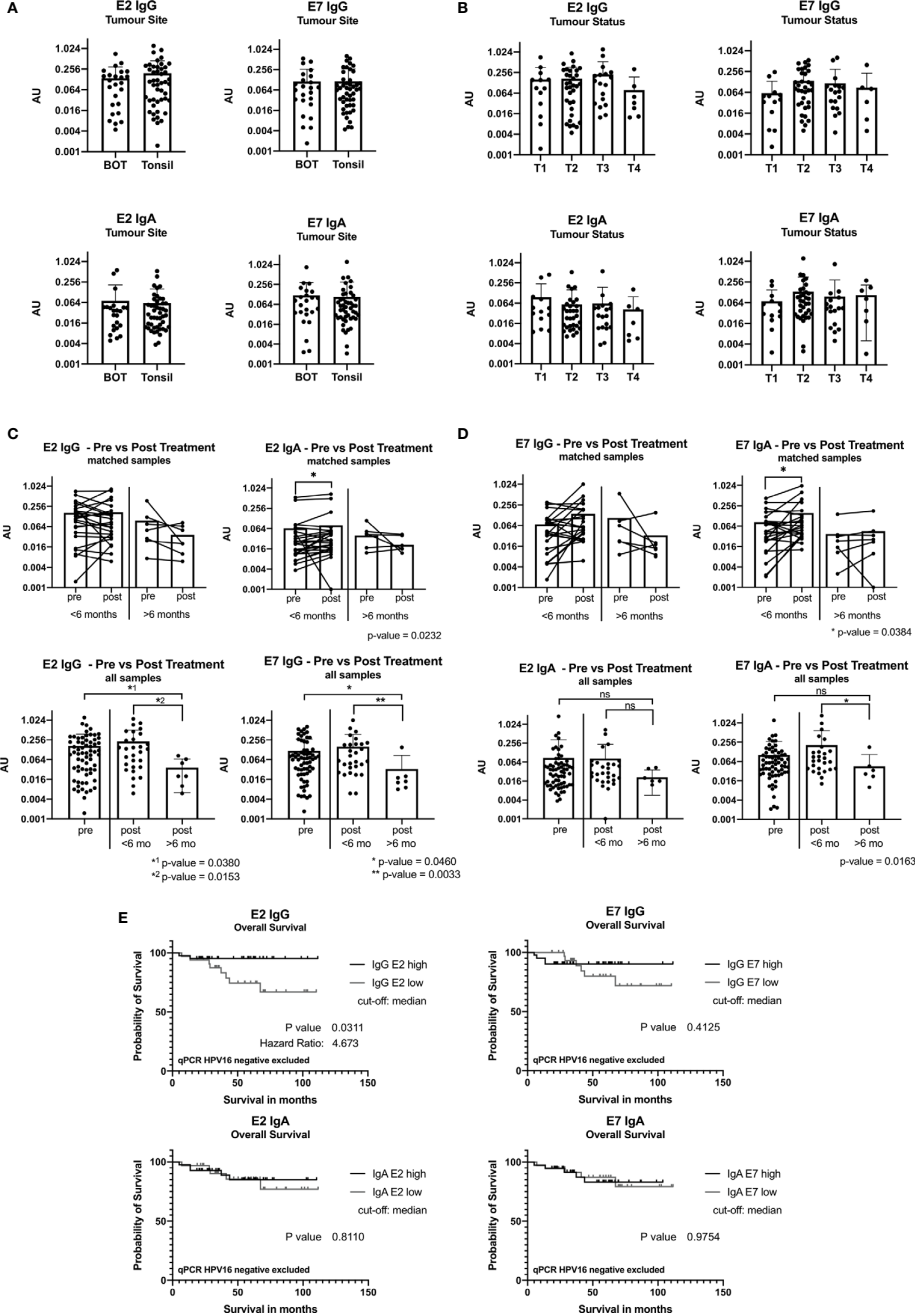
ELISA results reported in arbitrary units (AU) from samples taken at baseline before treatment. **(A)** Bar graph showing AU values for the tumor site base of tongue (BOT) and tonsil for the antibody classes IgG and IgA. **(B)** Bar graph displaying AU values depending on the tumor size [small tumor size (T1) and advanced tumor status (T4)]. **(C)** Graph showing ELISA AU values of pre- and post-treatment samples for IgG. The top two figures show the paired E2 IgG data grouped by time in months when the second sample was taken after completion of treatment, less than 6 months (<6 months) and more than 6 months (>6 months). The bottom graphs display all pre-treatment and post-treatment samples and are plotted separated by their second sample date. Significance levels are shown for E2 IgG and E7 IgG (p<0.05). **(D)** Graphs showing the ELISA results for IgA with a statistically significant increase in the group with sample <6 months after treatment end (E2: p=0.0232, E7: p=0.0384). (ns, not significant; *: p≤0.05, **: p≤0.01). **(E)** Kaplan-Meier graph of overall survival of 74 patients (n=3 qPCR HPV16 negative excluded) grouping in IgG E2 low (n=33) and IgG E2 high (n=41) using median antibody values. The IgG E2 high group shows a significant better overall survival than the low AU value group (OS after 5 years: 95% vs 74%, p=0.0.311). E7 IgG shows a not statistically significant difference of the overall survival.

Antibody levels were not related to the location of the primary tumor (**Figure 3A**), nodal status or primary treatment strategy (not shown). **Figure 3B** shows that T2 and T3 tumors appear to have higher levels of E2 and E7 IgG, and E7 IgA antibodies, but these differences did not reach significance.

### IgA Antibody Levels Are Increased Early After Treatment

We compared pairs (n=32) with an interval of ≤6 months or >6 months between samples to understand the post-treatment kinetics. IgA antibody levels against E2 and E7 increased within the first 6 months post-treatment (p=0.0232 and p=0.034, respectively), while this was not seen for IgG (**Figures 3C, D** **)**.

We compared all baseline samples (n=67) against all post-treatment samples (n=36). IgG and IgA antibody levels remained stable ≤6 months after treatment. A significant decrease in IgG antibody levels >6 months after treatment was seen (**Figures 3C, D** **)**. This was also the case for anti-E7 IgA antibodies, but it did not reach significance.

### Patients With High Levels of Anti-E2 IgG Have a Better Overall Survival

Using ELISA and follow-up data from 77 patients, we performed Kaplan-Meier survival analysis. When the cohort was divided into E2 IgG high vs E2 IgG low, according to the median AU values, patients with high E2 IgG antibody levels showed a significantly longer survival than those with low levels (p=0.0321). Exclusion of HPV^neg^ qPCR cases did not change the survival analysis, on the contrary, it increased the significance slightly to p=0.0311 (**Figure 3E**). A similar trend was shown for E7 IgG, but we did not detect a link between IgA antibody levels and outcome (**Figure 3E**).

### High HPV16 DNA Levels and RNA Expression for E7 Are Correlated With Antibody Level

**Figure 4:**
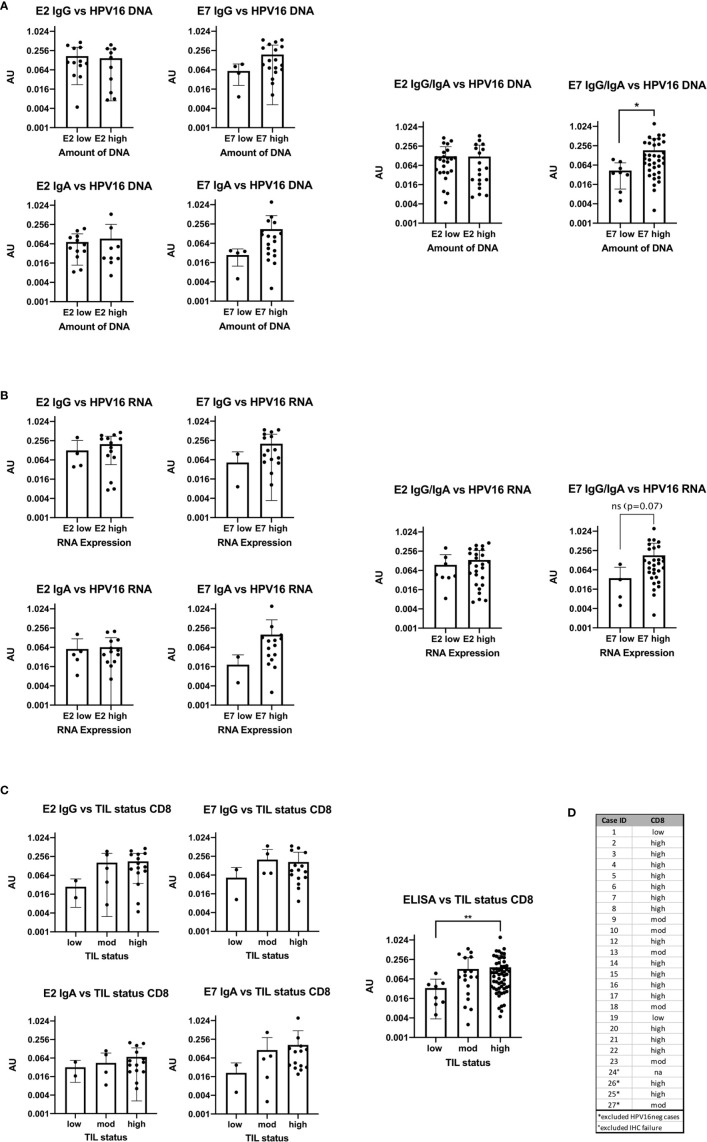
Bar plots showing the comparison of antibody levels with E2 and E7 expression levels on DNA, RNA and with the TIL status **(A)** High DNA expression level of E7 is accompanied with statistically significant higher AU values (p=0.0245) of E7 IgG in the serum **(B)** the RNA expression level and ELISA antibody units are congruent to the DNA results, but not statistically significant (p=0.07). **(C)** Patients with high or moderate TIL status assessed by CD8 have higher AU values in the serum for E2 and E7 IgG than patients with TIL^low^ tumors. The same trend is seen for E2 and E7 IgA. Combining all ELISA data to a summarized seropositivity the TIL^high^ HNSCC show significantly higher AU values than the low. (**: p≤0.01). **(D)** Table showing the case numbers and their TIL status for CD103 and CD8.

AU values were plotted against the amount of E2 and E7 DNA and RNA after classifying them into a low value (40-ΔΔCt <30) or high value (40-ΔΔCt ≥30) group (DNA: **Figure 4A**, RNA: **Figure 4B**). The cut-off was based on the rounded average 40-ΔΔCt values of 30.42 on DNA level. Detection of more E2 DNA or transcripts did not relate to higher serum levels of anti-E2 IgG or IgA antibody. However, E7 DNA and RNA transcripts were associated with a higher AU value for anti-E7 IgG and IgA, although the effect was not statistically significant, most likely due to low case numbers. Grouping the ELISA results showed that high amounts of DNA were significantly correlated with high AU values (p=0.035).

### TIL Status Has an Influence on Antibody Levels Against HPV16 E2 and E7

The TIL status of patients was determined using IHC staining specific for CD8^+^ cells and categorized into high, moderate and low as previously published (13). In the HPV16^pos^ qPCR cohort (n=24) 15 patients had a high TIL count for CD8. The rest showed a moderate or low TIL count (**Figures 4C, D** **)**.

AU were plotted in the three TIL categories according to the number of CD8^+^ cells (**Figure 4C**). Due to the small numbers in the low expression group we combined all ELISA data (E2 IgG, E2 IgA, E7 IgG, and E7 IgA) and compared the overall seropositivity with the TIL status. Significant more circulating antibodies in TIL^high^ tumors were found (p=0.0097).

## Discussion

Several diagnostic approaches for HPV16 detection are described. The assessments include HPV-PCR, HPV-*in situ hybridization* (ISH) and the hybrid-capture HPV DNA Test which can be used to detect a whole group of high-risk and low-risk HPV types (39). Hitherto, only few methods for the detection of HPV16 in FFPE material have been published (40). Systematic assessment of the HPV16 genome, quantifying all genes separately, had not yet been undertaken. The primer pairs we used in the qPCR assay for HPV16 detection were rigorously tested to be specific for each HPV16 gene, without cross reactivities to other high-risk HPV types, or other human viruses. The assay specificity was also supported by using the viGen bioinformatic pipeline (31) to detect HPV16 genes, mapping the RNA-sequencing data onto HPV16 genome. This separate analysis using different starting material revealed the same findings and confirms the validity of our qPCR assay.

Evaluation of all HPV16 genes in OPSCC with qPCR revealed highly correlated amounts of HPV16 gene DNA with the lowest values for E5 and E7. Intriguingly, we could detect all HPV16 genes in most cases. E5 expression displayed the lowest correlation with other HPV16 genes and was absent in case 24. E5 is composed of 83 amino acids, making it the smallest of all HPV16 genes. E5 has been shown to be necessary for early cancer development and can be lost during the course of cancer development (41). Our case is likely an example of this process. We identified 3 HPV^neg^ cases in our cohort (n=27), which expressed high levels of p16 protein. This is approximately 11% and is in the lower range of published percentages of 15%–20% for p16^+^ but HPV-ISH^neg^ HNSCC (7).

The 40-ΔΔCT values for DNA differ from those identified for RNA expression, reflecting variable transcriptional activity between cases. The late genes L1 and L2 are both transcribed from the late promotor and are only expressed in the surface of the stratified squamous epithelium, while in the basal cells only early genes are transcribed (42). The assembly and dissemination of HPV16 from the superficial epithelial cells happens after late antigen expression. However, in the case of tumor formation, viral assembly is not critical once transformation has occurred and hence, the late genes are likely redundant. Intriguingly, the late antigens are still transcribed into RNA in 58% of cases for L1, 53% for L2 and 37% expressed both. This finding underpins the possibility of complete viral reassembly in more than one third of cases, supporting the risk of viral transmission between partners after the cancer has been established (43). If true, this has important social implications for the patient and their partner(s). This question should be evaluated formally in prospective work to assess whether a prophylactic vaccine may be indicated for partners and patients.

We describe two cases where E7 DNA is detected, but not transcribed. Both oncogenes E5 and E6 however are transcribed in those cases. It is not clear if E7 was expressed during cancer initiation, and then lost during cancer growth, or if there is an immunological mechanism (one case was TIL^high^, the other TIL^low^). In contrast to published data, we could detect E2 RNA at the same time as RNA for E6 and E7 (9) suggesting that E2 does not necessarily exert inhibitory transcriptional control over E6/E7 expression (8).

It has been reported that patients with HPV16^pos^ OPSCC show increased levels of E6 and E7 antibodies in the blood, independent of the viral load (16). However, we find a correlation between E7 DNA and RNA expression and higher serum levels of IgG and IgA antibodies against E7, but not E2: thus, individual genes of HPV16 appear to be differentially expressed, transcribed and could be differentially immunogenic. Therefore, a detailed analysis on a gene by gene basis is important for being able to interpret immunological data.

E2 has previously been reported to be associated with a greater cytotoxic T lymphocyte response compared to E7 (44). We did not directly assess T cell reactivity in our cases; the humoral responses we observe however raise the possibility that T helper cells are less activated in response to E2 than E7, and therefore lead to less B cell activation and antibody production. The relationship between HPV16 gene expression and antigenicity requires further investigation. This would also have important implications for vaccine development.

The presence of viral antigen leads to the activation of immune responses aimed at clearing the infection. This clearance requires both T and B cell activation. T cells activated against HPV antigens are able to recognize the intracellular virus through presentation of viral peptides in MHC molecules, and this has been shown by the presence of virus-specific T cells in HPV^pos^ HNSCC (45). These T helper cells activate B cells to produce specific antibodies which can then be detected in the serum of patients e.g. by ELISA. ELISA is a rapid and inexpensive assay which can give additional information about the patients’ prognosis beforehand and could be a useful additional diagnostic tool.

Antibodies directed against HPV early antigens have been proposed as a prognostic biomarker before and after removal of the tumor (46). If treatment is successful it would remove the source of HPV antigen, limiting B cell responses. Therefore, following effective CRT or complete surgical resection of all disease, a significant decrease or loss of antibodies over time is expected. Continued detection of antibodies may indicate either residual tumor (post-CRT) or distant metastasis (post-complete surgical resection). 15%–20% of HPV^pos^ patients die from residual or recurrent disease within 2-years and a simple blood based biomarker would be clinically relevant (47). While pre- and post-surgery levels of E6 antibody have been described as a prognostic indicator of recurrence (48), pre and post-treatment levels of antibody to E7 appear to serve as a biomarker. In our study, the pre- and post-treatment antigen levels were different in the two groups sampled at different times (<6/>6 months). However, we could not assess the relevance of antibodies as a biomarker of recurrence in our cohort as no recurrences occurred during the period of sample collection.

We saw stable antibody levels for E2 IgG before treatment and in serum samples taken in the first 6 months after treatment, while there was a slight increase in anti-E7 IgG; after that timepoint antibody levels decreased. This finding is consistent with the published data by Fakhry et al. (48). For IgA, in the first 6 months after surgery, we saw increasing antibody levels in the blood, but again after 6 months, these levels decreased. Therefore, even after the tumor is completely removed, antibodies persist in the blood. As the half-life of immunoglobulins is much less than 6 months, these findings must mean that HPV16-reactive B cells/Plasma cells may persist in lymphatic structures, for example the bone marrow. Nonetheless, these B cells must be relatively short lived as, following tumor removal, we find that after 6 months antibody levels decline. As expected, the kinetics for E6 and E7 DNA are different and decrease rapidly after treatment in oral rinse samples in contrast to the antibody responses (49), demonstrating that both tests offer different biological insights in to the success of treatment.

Consistent with published data (17, 50) we confirmed better survival for OPSCC patients with IgG responses to the E2 gene it is thought that the antigenic determinant is located at the N terminal region of E2 (18). A fascinating new observation from our study is that the presence of IgA antibodies does not appear to link to survival benefit. This is intriguing, as IgA responses have been accused of dismantling adaptive T cell responses in human liver cancer (51). Formal study of the biological function of IgA^+^ B cells and plasma cells is needed to understand the underpinning biology. Consistent with the published data, there was no significant survival benefit seen for E7 antibody seropositivity, although a trend emerged after longer follow-up.

Generally, patients with HPV^pos^ OPSCC have a better outcome than HPV^neg^ patients, but those HPV^pos^ with low TIL status have the same poor outcome as HPV^neg^ OPSCC (13). Patients with immune-cold tumors (TIL^low^) had less antibody production, while immune-hot tumors (TIL^high^) were associated with greater antibody production. Our data is consistent with the observation that B cells and T cells are abundant in the same cancers (52), the antibody levels demonstrate the functional link between these cell populations, most likely anatomically located together in tertiary lymphatic structures in the cancer microenvironment. Importantly, the circulating antibody levels may be a potential biomarker to stratify patients in clinical trials evaluating immunotherapies.

Caveats for generalizing our data include the small size of our qPCR cohort, as some samples were too low-quality for further analysis. This might also be a limitation in using FFPE material: RNA is less stable than DNA and consistent with this is that technical failures occurred in the older FFPE blocks.

In our serum sample cohort, the selection criteria were based on p16, as the commonly available surrogate marker for HPV-driven disease. This may have biased case choice and going forward, we would use the qPCR for DNA in combination with p16 IHC as the more robust tool for identifying HPV16^pos^ tumors.

The ELISA assay had initially been planned to include antibodies against E2, E6 and E7. However, we did not successfully express E6 protein. Nevertheless, including E6 and E5 in future research could unfold additional insights in antibody responses to those oncogenic HPV16 antigens. ELISA findings have to be confirmed prospectively in a larger HPV^pos^ OPSCC cohort with standardized and consecutive serum sample collection. This would validate our results and improve the understanding of HPV16 expression at genomic and transcriptomic levels, as well as antibody responses and their clinical impact.

## Data Availability Statement

The original contributions presented in the study are publicly available. This data can be found here: https://www.ncbi.nlm.nih.gov/geo/query/acc.cgi?acc=GSE160008.

## Ethics Statement

The studies involving human participants were reviewed and approved by UK Medical Research and Ethics Committee and by institutional approval at Southampton University Hospitals NHS Foundation Trust, Southampton, UK. The patients/participants provided their written informed consent to participate in this study.

## Author Contributions

AW designed the study. AW did the experimental work, analyzed data, and wrote the manuscript. EC co-wrote the manuscript. JT undertook bioinformatic analyses of the HPV16 alignment and RNA-sequencing. OW and LC provided technical and experimental support. GT contributed pathological supervision and supervised the IHC. OA contributed to the method development of the ELISA assay. EK, PF, and SL contributed to writing and ordering of the manuscript. CO designed the study, supervised the experimental work and data analysis and co-wrote the manuscript. All authors contributed to the article and approved the submitted version.

## Funding

AW is funded by a research fellowship of the German research association (Deutsche Forschungs Gemeinschaft, DFG; research fellowship # WI 5255/1-1:1). OW is funded by a Cancer Research UK Centre’s Network Accelerator Award Grant (A21998). SL is funded by DFG research training group (GRK-2254). EC, LC, and CO have funding from Southampton Hospitals charity donations. RNA-sequencing was funded by Transgene.

## Conflict of Interest

Author KB was employed by company Transgene SA.

The remaining authors declare that the research was conducted in the absence of any commercial or financial relationships that could be construed as a potential conflict of interest.
