# Supplementary material for: Correlation of HPV16 Gene Status and Gene Expression With Antibody Seropositivity and TIL Status in OPSCC
Source: Front Oncol. 2021 Jan 26;10:591063. doi: 10.3389/fonc.2020.591063 (PMC7871909; doi:10.3389/fonc.2020.591063)
Supplement: Supplementary file 1 [file DataSheet_1.docx]

Supplementary Material

# Supplementary Data

# Supplementary Figures and Tables

## Supplementary Figures

**Figure S1:** The Kaplan-Meier graph shows the overall survival with a median follow-up time of 4 years and 5 months and an overall survival of 94% after 2 years and 85.3% after 5 years. There is no difference in survival regarding the gender, tumour site or nodal status. A trend towards worse survival in the >70 years old patients is observed. Outcome deteriorates with increasing tumour T and overall TNM stage. The grouping by different treatment shows for CRT/RT and surgery no strong difference. But the group with failed CRT/RT treatment show worse survival.

**Figure S2**: Correlation (including HPV16 ^neg^ cases) of the qPCR results of all HPV16 genes including all four E5 primer pairs. The left heatmap shows the r-values and the right the corresponding significance levels of correlation. No correlation for the E5 Paolini et al. primer is shown.

**Figure S3**: Heatmap showing the r-values for correlation of the ELISA antibody units for E2, E7 with IgG and IgA. All comparisons are statistically significant and have the highest r-values between E2 IgA and E7 IgA, followed by E2 IgG and E7 IgG.

## Supplementary Table

Supplementary Table 1: Summary of samples which were available for the performed experiments.

| Samples |  | n= | Percentage |
| --- | --- | --- | --- |
| Patients | All | 77 | 100% |
| Serum Samples | Pre treatment | 73 | 95% |
|  | Post treatment | 40 | 52% |
|  | Matched pre and post | 36 | 47% |
|  |  |  |  |
| qPCR (FFPE) |  | 27 | 35% |
|  |  |  |  |
| RNA Sequencing  (snap frozen tissue) |  | 4 | 5% |
|  |  |  |  |
| IHC (FFPE) |  | 27 | 35% |
